# Impact of Two Pretreatment Methods on the Chemical Profile of Still-Bottom Water and the Antioxidant Activities of Hydrosol and Still-Bottom Water from *Citrus × aurantium ‘Daidai’* and *Citrus × aurantium* L. Dried Flower Buds

**DOI:** 10.3390/plants15172583

**Published:** 2026-08-25

**Authors:** Li Hao, Ting Li, Tingting Chen, Liqin Yin, Yan Wan, Yayu Zhang, Huan Wang, Yi Zhang, Yanli Xia

**Affiliations:** 1 College of Food and Biological Engineering, Chengdu University, Chengdu 610106, China; haoli@cdu.edu.cn (L.H.); 2025101026@stu.sicau.edu.cn (T.L.); 212021095135009@cdu.edu.cn (T.C.); yinliqin@cdu.edu.cn (L.Y.); yanwan@cdu.edu.cn (Y.W.); zhangyayu@cdu.edu.cn (Y.Z.); wanghuan@cdu.edu.cn (H.W.); 2 College of Agronomy, Sichuan Agricultural University, Chengdu 611130, China; 3 Chengdu Institute of Biology, Chinese Academy of Sciences, Chengdu 610041, China

**Keywords:** *Citrus × aurantium*, flower buds, still-bottom water, hydrosol, flavonoid glycosides, chemical antioxidant activity

## Abstract

*Citrus × aurantium ‘Daidai’* (*Citrus aurantium* L. var. amara Engl.) is a sour orange cultivar whose dried flower buds are listed in the Chinese national catalogue of substances that serve both as food and as medicine, whereas the dried flower buds of the ordinary species *C. × aurantium* L. lack this dual recognition. Although the essential oil and hydrosol of these buds have been investigated, the still-bottom water remaining after hydrodistillation is routinely discarded and its chemical composition remains largely unexplored. In this study, we compared the chemical profiles of still-bottom water obtained from the dried flower buds of *C. aurantium* L. var. amara Engl. (CAVAF) and *C. × aurantium* L. (CALF) after two pretreatments (simple soaking vs. ultrasonic-microwave synergistic treatment) and evaluated the chemical antioxidant activities of both the hydrosol and the still-bottom water. HPLC-Q-TOF-HRMS identified 215 compounds across the four water samples. The major constituents were neohesperidin (13.18–39.66%), blumeatin (9.78–10.25%), narirutin (13.46–18.01%), and α-D-(6-O-Sinapoyl)-glucopyranosyl(1→22)-β-D-(3-O-sinapoyl)-fructofuranose (7.46–8.57%). Relative to CALF, the CAVAF samples contained a large number of annotated compounds and higher relative amounts of several flavonoids and limonoids (up to nine-fold) in the still-bottom water. Higher relative abundances of the principal flavonoid glycosides were observed after ultrasonic-microwave pretreatment compared with soaking. The hydrosol of both varieties showed only modest radical scavenging activity and weak reducing power. In contrast, the still-bottom water exhibited clear DPPH, ABTS, and hydroxyl radical scavenging, with half-maximal inhibitory concentrations ranging from 0.1901 to 4.873 mg/mL, although reducing power remained limited. These findings suggest that botanical variety and pretreatment method may influence both the metabolite profile and the in vitro radical scavenging behavior of the distillation by-products. The results supply chemical compositional support for the food-medicine homology status of CAVAF and indicate that still-bottom water is a recoverable source of potential polar antioxidants that is currently under-utilized.

## 1. Introduction

*Citrus × aurantium* L., commonly known as sour or bitter orange, is an evergreen member of the Rutaceae that is widely cultivated in tropical and subtropical regions [1]. In addition to its long-standing use as a rootstock for other *Citrus* species [2], the plants supply fruits, flowers, and flower buds that find applications in functional foods, cosmetics, and traditional medicine [3]. In the Chinese Pharmacopoeia, both the mature fruit (*Aurantii fructus immaturus*) and the immature fruit (*Aurantii fructus*) are recognized as official drugs [4,5]. The fragrant white flowers, meanwhile, have a well-documented history of culinary use in the Mediterranean region and medicinal use in east Asia [6]. Among the various Chinese varieties, *Citrus × aurantium ‘Daidai’* (*Citrus aurantium* L. var. amara Engl., abbreviated as CAVA), occupies a special position. Its dried flower buds are the only *Citrus* flower buds currently listed in the national catalogue of substances that may be used both as food and as medicine, while the dried flower buds of the ordinary species (*C. × aurantium* L., abbreviated as CAL) lack this dual recognition.

Phytochemical investigations of the flowers have revealed a rich array of polyphenols, flavonoids, alkaloids, and terpenes that collectively account for a range of reported biological activities, including antidepressant, antimicrobial, anti-inflammatory, antioxidant, and antihyperlipidemia effects [6,7,8,9,10]. The flavonoid fraction of CAVAF, dominated by hesperidin, naringin, and neohesperidin, has been shown to contribute substantially to the observed antioxidant capacity [11]. 

Industrial recovery of the essential oil from these flower buds is performed by hydrodistillation and simultaneously generates two aqueous by-products, the hydrosol (the condensed aqueous distillate) and the still-bottom water that remains in the distillation flask [1]. The latter fraction constitutes the larger volume and is almost invariably discarded, even though it retains the bulk of the non-volatile, highly polar secondary metabolites. The yield and composition of these metabolites are strongly influenced by the conditions applied before distillation. Various pretreatments, including simple immersion, ultrasound, microwave irradiation and enzymatic treatment, have been shown to improve both yield and chemical quality relative to hydrodistillation alone [12,13,14,15,16]. Ultrasound-assisted extraction relies on acoustic cavitation to disrupt cell walls and enhance solvent-compound contact, while microwave-assisted extraction employs electromagnetic energy to generate rapid internal heating and pressurization, thereby accelerating the release of intracellular solutes without extensive thermal degradation [14,17]. When these two techniques are combined, they offer a green and efficient alternative that shortens processing time, reduces solvent consumption and generally increases the recovery of bioactive constituents [18]. Despite these advantages, the comparative effects of conventional soaking versus ultrasonic-microwave synergistic pretreatment on the chemical profiles of still-bottom water, and on the antioxidant activities of both hydrosol and still-bottom water from CAVAF and CALF, have not been systematically examined. 

Given the food-medicine homology status of CAVAF and the traditional use of CALF, a side-by-side investigation of their distillation by-products would help to fill the knowledge gap concerning still-bottom water composition and provide a factual basis for the potential high-value utilization of these currently discarded resources, consistent with the principles of circular economy and sustainable biorefinery. In this study, we characterize and compare the chemical profiles of still-bottom water obtained from CAVAF and CALF under the two pretreatment protocols by means of HPLC-Q-TOF-HRMS, and we evaluate the antioxidant activities of the corresponding hydrosol and still-bottom water with DPPH, ABTS, hydroxyl radical scavenging, and reducing power assays. The results provide preliminary insights into how botanical variety and the pretreatment method may influence the composition and bioactivity of these by-products, thereby supplying chemical support for the food-medicine homology status of CAVAF and for the potential recovery of value from an otherwise wasted stream.

## 2. Results

### 2.1. Chemical Composition of Still-Bottom Water from CAVAF

The chemical constituents of the soaking-derived and ultrasonic-microwave-derived still-bottom water from CAVAF were characterized by HPLC-Q-TOF-HRMS. Total ion chromatograms (TICs) acquired in both positive and negative ion modes are shown in Figure S1, with detailed identification data listed in Tables S1–S4.

In positive ion mode, 138 compounds were annotated in the soaking-derived sample and 127 in the ultrasonic-microwave-derived sample. Flavonoids accounted for the largest proportion of relative peak area in both cases (60.07% vs. 61.21%), followed by glycosides (5.08% vs. 5.15%), phenols (4.93% vs. 4.83%), limonoids (4.29% vs. 3.85%), alkaloids (4.77% vs. 3.70%), and organic acids (3.23% vs. 3.21%). Together these six classes represented more than 80% of the total relative content. Neohesperidin was the most abundant constitutent in both samples (13.18% after soaking and 13.53% after ultrasonic-microwave treatment), followed by blumeatin (10.11% vs. 10.12%), α-D-(6-O-sinapoyl)-glucopyranosyl(1→2)-β-D-(3-O-sinapoyl)-fructofuranose (7.56% vs. 7.93%), narirutin (5.81% vs. 5.86%), and 2″-O-Glucosylisoswertisin (5.70% vs. 6.04%). Several additional compounds, including 2,7-dihydroxy-4-methoxyphenanthrene-2-O-glucoside, butein, oroxin B, limonin, daidzein-4′,7-diglucoside, and cimicifugic acid, exceeded 3% relative content under one or both pretreatments (Tables S1 and S3).

In negative ion mode, 87 compounds were annotated after soaking and 72 after ultrasonic-microwave treatment. Flavonoids again dominated the relative composition (79.21% and 82.06%). Neohesperidin remained the most prominent peak (31.82% and 38.11%), followed by narirutin (13.46% vs. 14.78%), α-D-(6-O-sinapoyl)-glucopyranosyl(1→2)-β-D-(3-O-sinapoyl)-fructofuranose (7.46% vs. 8.00%), neoeriocitrin (6.02% vs. 7.11%), and oroxin B (4.66% vs. 4.90%). Norbergenin exceeded 3% in both samples, whereas nelumboroside A did so only in the soaking-derived sample (Tables S2 and S4).

### 2.2. Chemical Compounds of Still-Bottom Water from CALF

The still-bottom water obtained from CALF was examined under identical analytical conditions (Figure S2 and Tables S5–S8).

In positive ion mode, 146 compounds were annotated after soaking and 120 after ultrasonic-microwave treatment. Flavonoids again constituted the major class (59.61% and 61.48%). Neohesperidin was the leading constituent (13.99% vs. 15.78%), followed by blumeatin (9.78% vs. 10.25%), α-D-(6-O-sinapoyl)-glucopyranosyl(1→2)-β-D-(3-O-sinapoyl)-fructofuranose (8.05% vs. 8.57%), narirutin (6.41% vs. 7.10%), and 2″-O-Glucosylisoswertisin (5.10% vs. 5.43%). Limonin and cimicifugic acid exceeded 3% only in the soaking-derived sample, while (2S)-5,7-dihydroxy-6-methoxyflavanone-7-O-β-D-glucopyranoside reached this level only after ultrasonic-microwave treatment (Tables S5 and S7).

In negative ion mode, 88 compounds were annotated after soaking and 64 after ultrasonic-microwave treatment. Flavonoids accounted for 73.69% and 82.13% of the relative peak area. Neohesperidin was the most abundant (28.18% and 39.66%), followed by narirutin (14.51% vs. 18.01%), α-D-(6-O-sinapoyl)-glucopyranosyl(1→2)-β-D-(3-O-sinapoyl)-fructofuranose (8.21% vs. 7.98%), neoeriocitrin (6.96% vs. 7.05%), norbergenin (6.81% vs. 3.88%), and oroxin B (5.35% vs. 4.37%). Nelumboroside A (3.18%) and cimicifugic acid C (3.01%) exceeded 3% only in the soaking-derived sample (Tables S6 and S8).

### 2.3. Overall Comparison of the Four Still-Bottom Water Samples

**Figure 1:**
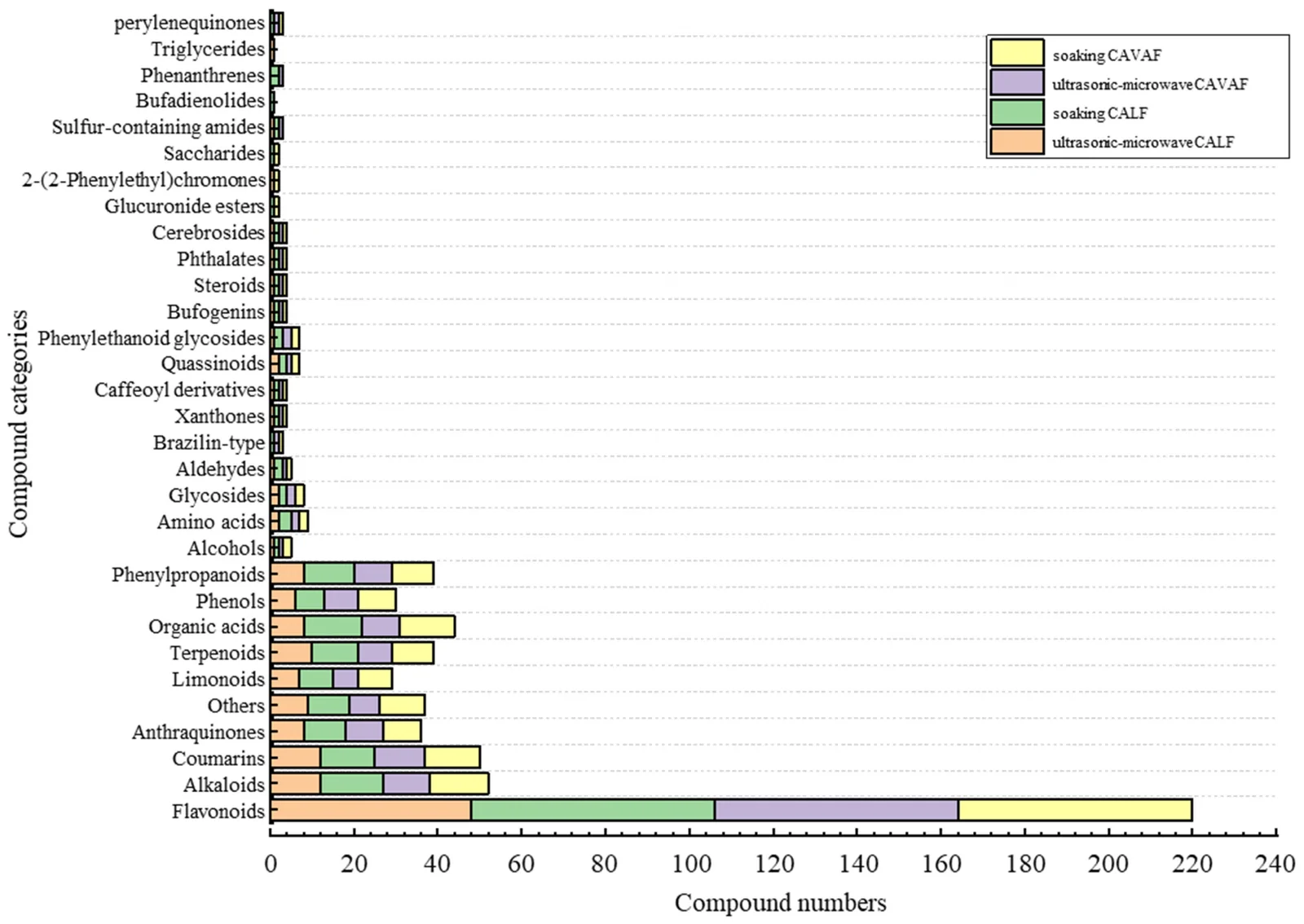
Comparison of the number of chemical compounds detected in the four still-bottom water samples.

A total of 215 unique compounds were annotated across the four samples. The numbers detected in each sample were 175 (soaking CAVAF), 156 (ultrasonic-microwave CAVAF), 183 (soaking CALF), and 146 (ultrasonic-microwave CALF). The principal compound classes included flavonoids, alkaloids, coumarins, organic acids, phenylpropanoids, terpenoids, anthraquinones, phenols, and limonoids (Figure 1 and Tables S1–S8). 

**Figure 2:**
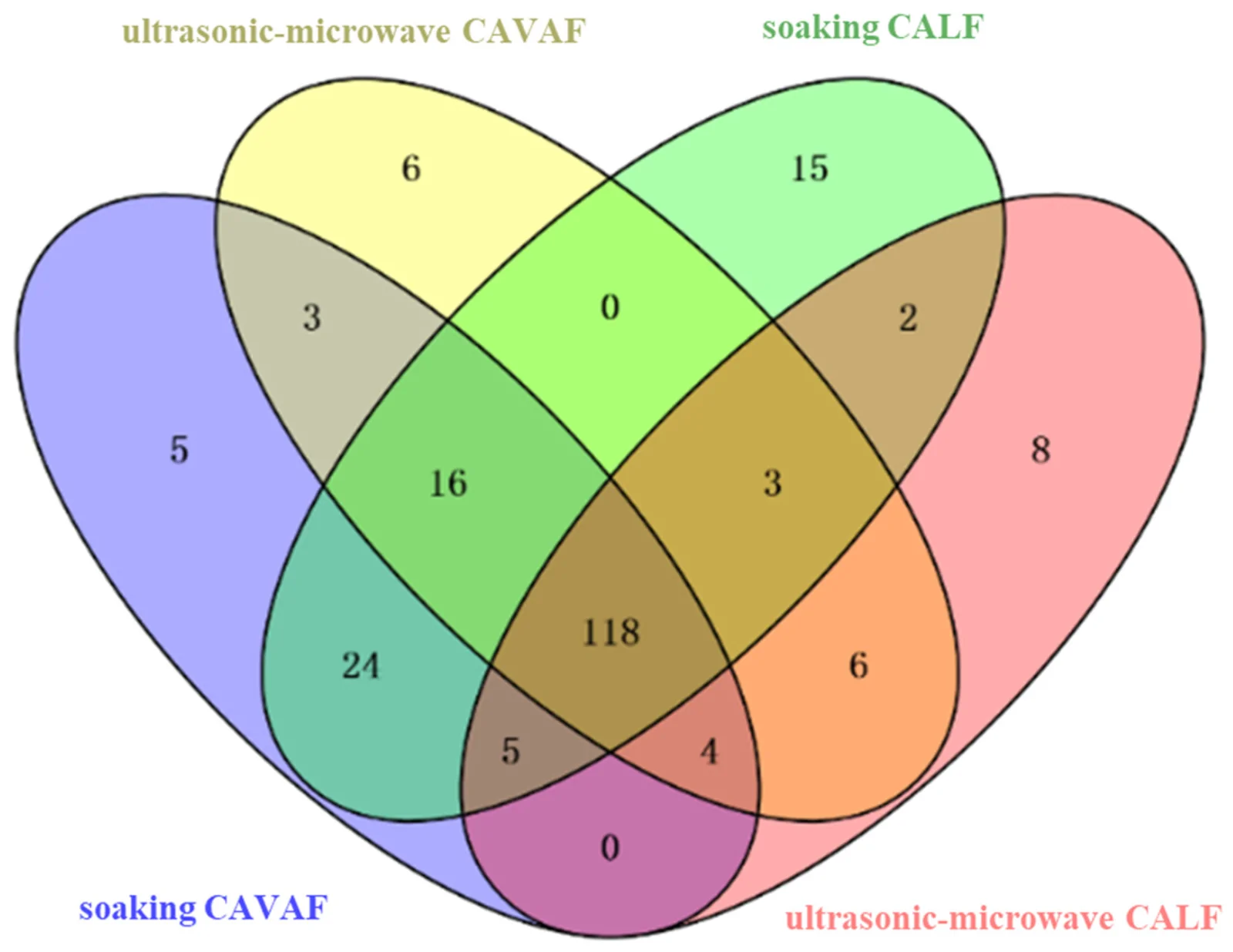
Venn diagram showing the distribution of chemical compounds identified in still-bottom water from CAVAF and CALF under the two pretreatments.

Under the same pretreatment, the CAVAF samples tended to yield a larger number of annotated compounds than the corresponding CALF samples. Soaking also retained a broader metabolite set (175 and 183) than ultrasonic-microwave treatment (156 and 146) for both varieties. Of the 215 compounds, 118 were common to all four samples (Figure 2 and Table 1). CALF contributed a greater number of unique compounds (25) than CAVAF (14). Soaking also produced more unique compounds (5 and 15) than ultrasonic-microwave treatment (6 and 8) (Figure 2 and Table 2). 

**Figure 3:**
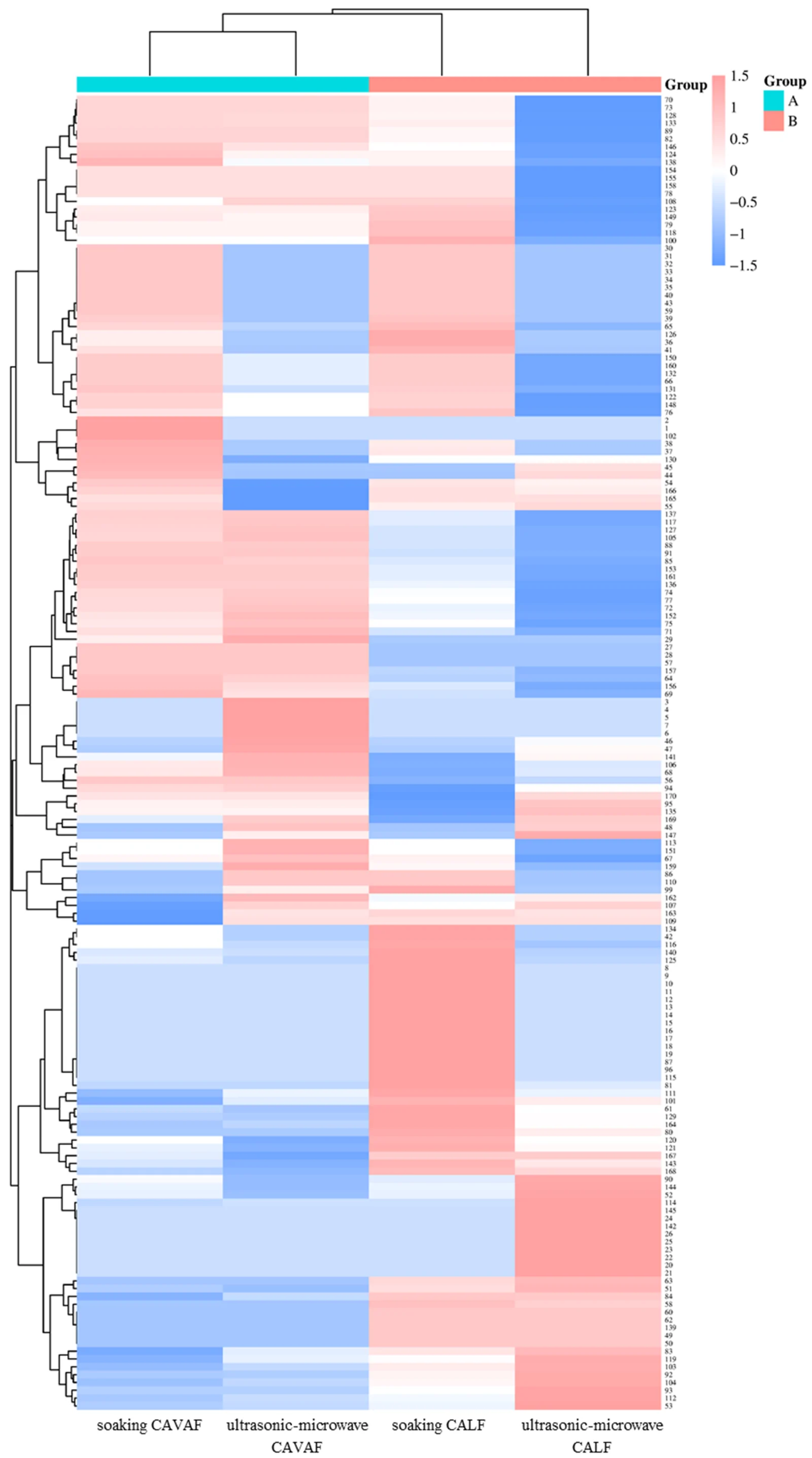
Heatmap of chemical compounds detected in still-bottom water under positive ion mode. Compound numbers are listed on the right, corresponding names are given in Tables S1, S3, S5 and S7. Red and blue indicate relatively high and low abundance, respectively.

**Figure 4:**
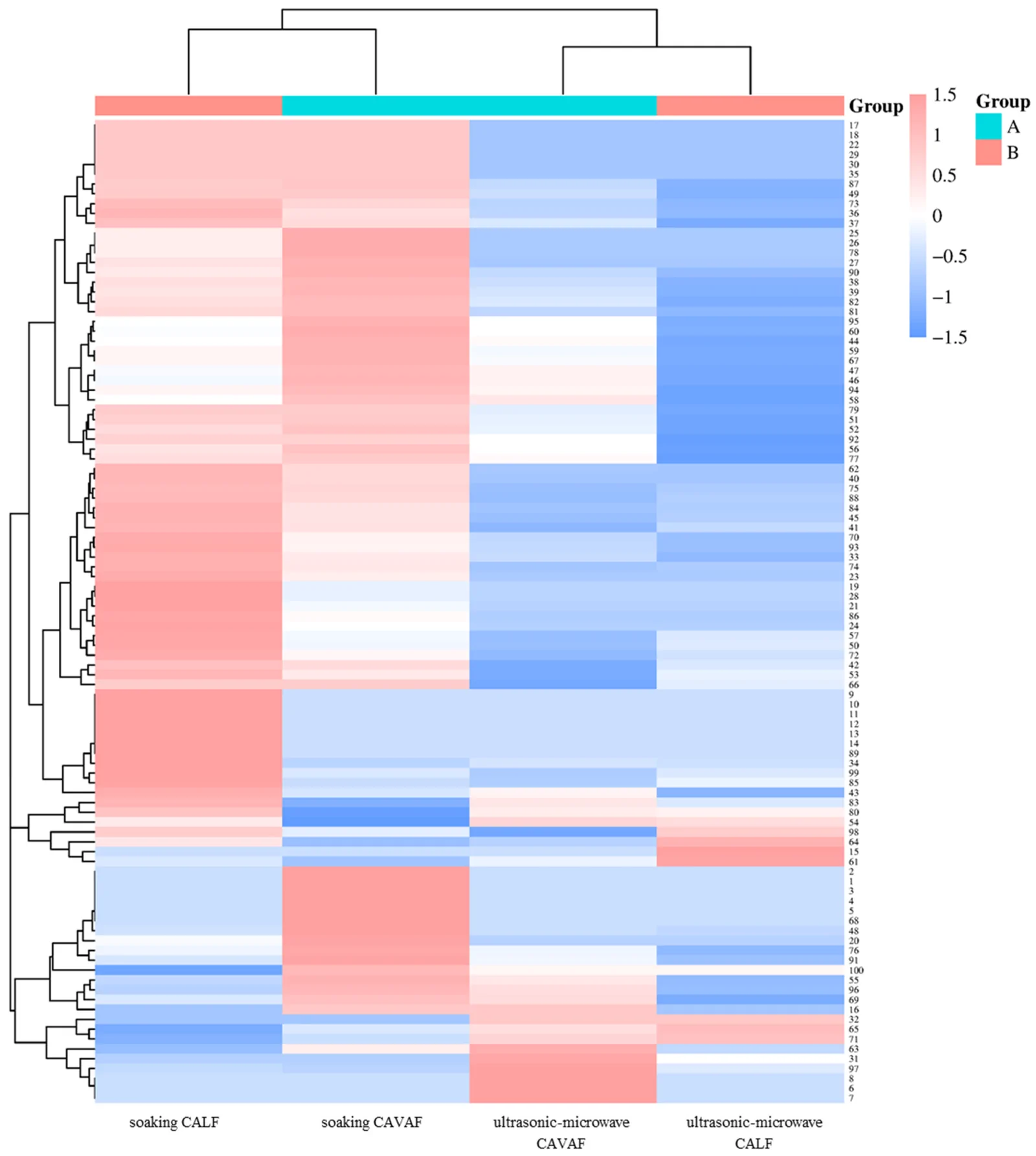
Heatmap of chemical compounds detected in still-bottom water under negative ion mode. Compound numbers are listed on the right, corresponding names are given in Tables S2, S4, S6 and S8. Red and blue indicate relatively high and low abundance, respectively.

Cluster analysis of relative peak areas indicated consistent differences between the two botanical sources (Figure 3 and Figure 4). Positive ion mode detected a larger number of compounds (170) than negative ion mode (100). Under identical pretreatment conditions, CAVAF samples showed higher relative levels of oroxin B, 2″-O-Glucosylisoswertisin, cimicifugic acid, limonin, and nelumboroside A, whereas CALF samples displayed higher relative levels of narirutin, neohesperidin, and α-D-(6-O-sinapoyl)-glucopyranosyl(1→2)-β-D-(3-O-sinapoyl)-fructofuranose. Fold differences for certain minor components reached three- or four-fold in positive ion mode and seven- to nine-fold in negative ion mode (most notably for M-3′-O-β-D-glucopyranoside, parvifloroside B, and kuwanon L).

Ultrasonic-microwave pretreatment was associated with higher relative abundances of several major flavonoid glycosides compared with soaking. In positive ion mode, these included oroxin B, narirutin, blumeatin, (2S)-5,7-dihydroxy-6-methoxyflavanone-7-O-β-D-glucopyranoside, 2″-O-glucosylisoswertisin, neohesperidin, and α-D-(6-O-sinapoyl)-glucopyranosyl(1→2)-β-D-(3-O-sinapoyl)-fructofuranose. In negative ion mode, higher relative abundances were observed for neoeriocitrin, narirutin, and neohesperidin. At the same time, the relative levels for cimicifugic acid, limonin (positive ion mode), and nelumboroside A (negative ion mode) were lower after ultrasonic-microwave pretreatment.

**Figure 5:**
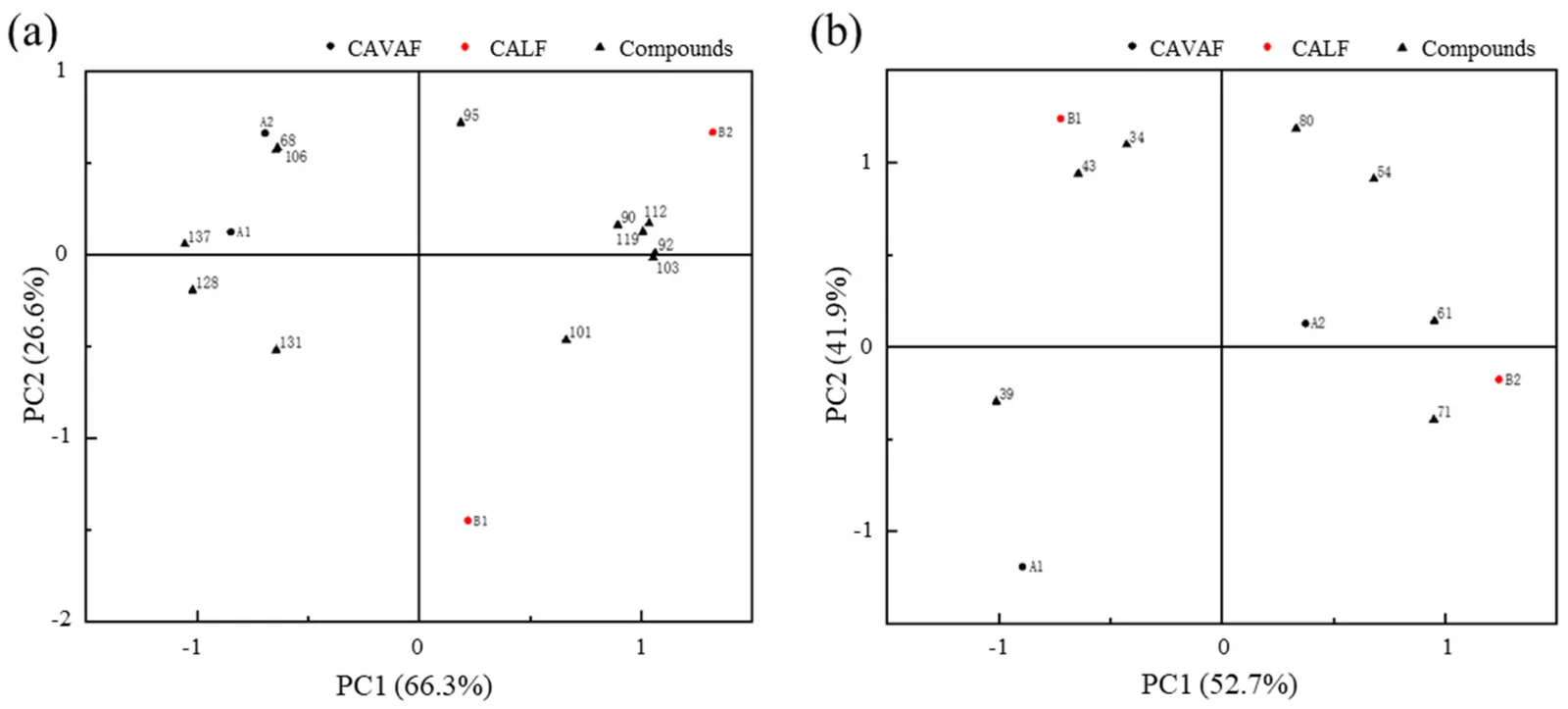
Principal component analysis of major compounds (relative content > 3%) in still-bottom water under positive (**a**) and negative (**b**) ion modes. A1: soaking CAVAF; A2: ultrasonic-microwave CAVAF; B1: soaking CALF; B2: ultrasonic-microwave CALF. Black triangles with numbers represent the major compounds, corresponding names are listed in Tables S1–S8.

Principal component analysis (PCA) was performed on the compounds whose relative content exceeded 3% (Figure 5) solely as an exploratory visualization of patterns within the present dataset. The first two principal components explained 92.9% of the total variance in positive ion mode (Figure 5a) and 94.6% in negative ion mode (Figure 5b). In positive ion mode, the separation between botanical varieties was more pronounced, and this distinction was mainly captured by PC1. In negative ion mode, the differentiation among pre-treatments became more evident, also primarily along PC1. Loading analysis indicated that narirutin, neohesperidin, nelumboroside A, α-D-(6-O-sinapoyl)-glucopyranosyl(1→2)-β-D-(3-O-sinapoyl)-fructofuranose, and neohesperidin contributed most strongly to the observed variance. Collectively, the PCA results indicated a clear separation according to botanical variety and pretreatment methods in this dataset.

### 2.4. Antioxidant Activities of Hydrosol and Still-Bottom Water

**Figure 6:**
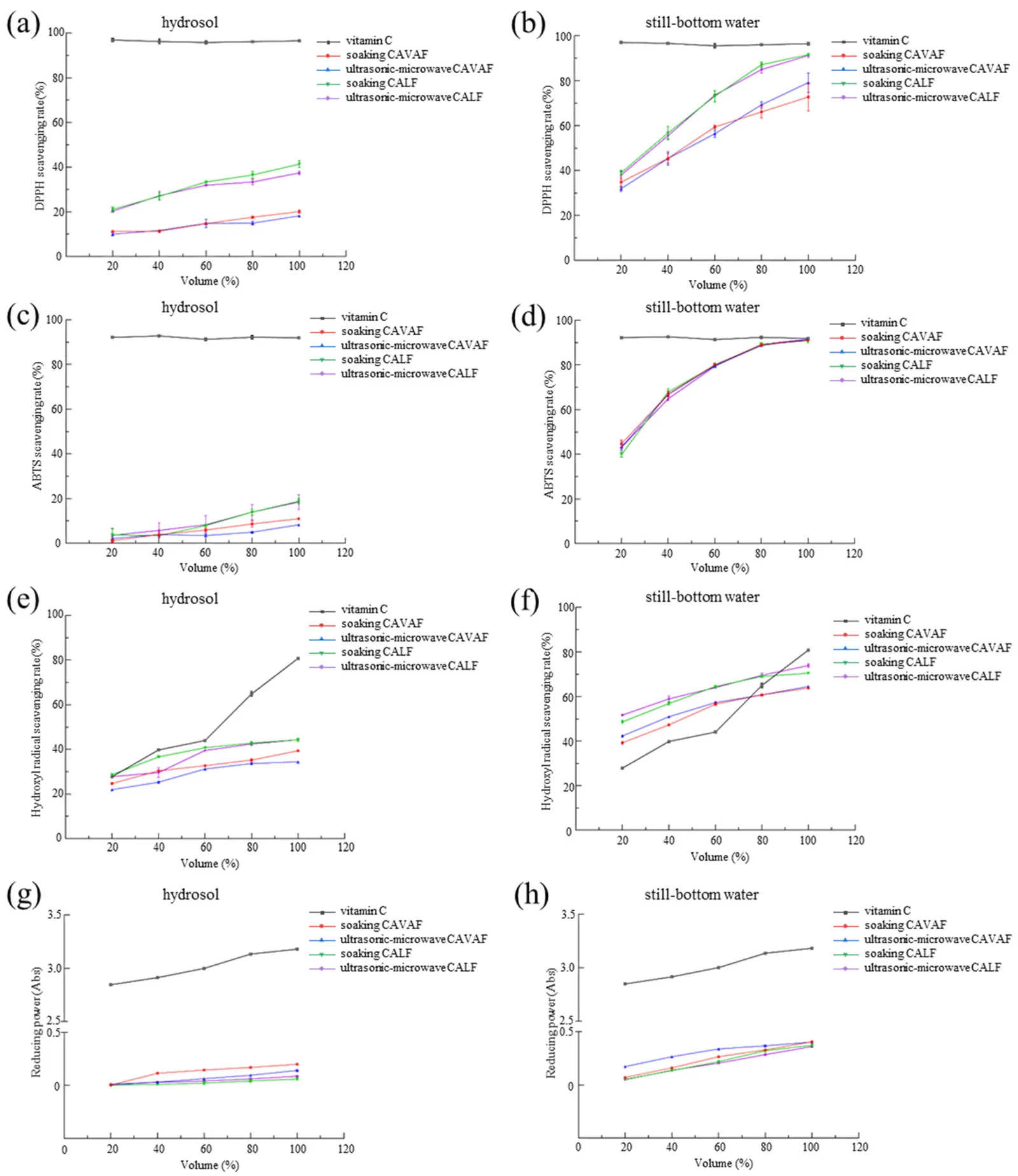
Antioxidant activities of hydrosol and still-bottom water derived from CAVAF and CALF. (**a**) DPPH scavenging by hydrosol. (**b**) DPPH scavenging by still-bottom water. (**c**) ABTS scavenging by hydrosol. (**d**) ABTS scavenging by still-bottom water. (**e**) Hydroxyl radical scavenging by hydrosol. (**f**) Hydroxyl radical scavenging by still-bottom water. (**g**) Reducing power of hydrosol. (**h**) Reducing power of still-bottom water. All measurements were performed in analytical triplicate. Error bars represent standard deviation.

Antioxidant activities of the hydrosol and still-bottom water were assessed by DPPH, ABTS, hydroxyl radical scavenging, and reducing power assay (Figure 6 and Table S9).

In the DPPH assay, scavenging activity of the four hydrosols increased with volume fraction, with the CALF hydrosol showing greater activity than the CAVAF hydrosol (Figure 6a). However, none of the hydrosols reached the half-maximal inhibitory concentration. Maximum scavenging rates at undiluted strength were 20.70% (soaking CAVAF), 18.20% (ultrasonic-microwave CAVAF), 41.27% (soaking CALF), and 38.29% (ultrasonic-microwave CALF). By contrast, the still-bottom water exhibited clear DPPH radical scavenging capacity, with half-maximal inhibitory concentrations of 4.779 mg/mL (soaking CAVAF), 4.873 mg/mL (ultrasonic-microwave CAVAF), 3.240 mg/mL (soaking CALF), and 3.338 mg/mL (ultrasonic-microwave CALF) (Figure 6b). At the highest concentration, the scavenging rates ranged from 65.69% to 92.25%.

A comparable pattern was observed in the ABTS assay. Hydrosol displayed only weak activity and did not reach the half-maximal inhibitory concentration, with maximum rates of 11.09% (soaking CAVAF), 8.46% (ultrasonic-microwave CAVAF), 20.01% (soaking CALF), and 22.12% (ultrasonic-microwave CALF) (Figure 6c). The still-bottom water, however, yielded half-maximal inhibitory concentrations of 2.384, 2.494, 2.683, and 2.588 mg/mL for the four samples, respectively (Figure 6d). All four still-bottom water achieved scavenging rates above 90% (90.71%, 91.27%, 90.09%, and 91.38%) at full strength.

In the hydroxyl radical scavenging assay, the hydrosol again displayed moderate activity (maximum 34.55–44.92%) without reaching half-maximal inhibitory concentration (Figure 6e). The still-bottom water was substantially more active, with half-maximal inhibitory concentrations of 0.4853, 0.4087, 0.1901 mg/mL for soaking CAVAF, ultrasonic-microwave CAVAF, and soaking CALF, respectively (Figure 6f). At full concentration, their scavenging rates ranged from 63.84% to 74.63%. Notably, within the intermediate volume fraction range (approximately 20–75%), all four still-bottom water samples showed higher activity than vitamin C under the assay conditions used.

Reducing power remained low for both fractions. At full strength, the maximum absorbance values for the hydrosol were 0.203, 0.149, 0.073, and 0.093, while those for the still-bottom water were 0.411, 0.407, 0.381, and 0.370, respectively.

Taken together, the hydrosol from both CAVAF and CALF exhibited only modest radical scavenging capacity. The still-bottom water, by contrast, showed clear activity in the DPPH, ABTS, and hydroxyl radical scavenging assays, although reducing power was limited. These observations are consistent with the presence of polar flavonoid glycosides retained in the residual aqueous fraction after hydrodistillation.

## 3. Discussion

The chemical contrast between hydrosol and still-bottom water follows directly from the physical principles of hydrodistillation. Volatile monoterpenes such as linalool, α-terpineol, and geraniol are readily carried over with steam and therefore dominate the hydrosol, whereas non-volatile, highly polar flavonoid glycosides and limonoids remain quantitatively in the distillation flask [19,20,21]. As a result, the same metabolites (neohesperidin, narirutin, neoeriocitrin, and related compounds) that are enriched in the still-bottom water may contribute to the observed radical scavenging activity.

The compositional differences recorded between CAVAF and CALF in this study are consistent with known genetic variation in secondary metabolite pathways within the genus *Citrus* [22]. Under both pretreatments, the CAVAF samples accumulated higher relative amounts of several flavonoids and limonoids, while the CALF samples accumulated higher relative levels of the major flavanone glycosides. These quantitative distinctions may supply candidate chemical markers that could assist in the authentication and quality control of the two materials. Earlier comparative analyses of closely related *Citrus* Chinese medicinal materials have already demonstrated substantial inter-species fluctuations in flavonoid content between *C. × aurantium* and *C. reticulate* [23], supporting the view that CAVAF and CALF, although both belonging to *C. × aurantium*, differ in the expression or regulation of key biosynthetic enzymes. 

The two pretreatment methods exhibited distinct characteristics. Ultrasonic-microwave pretreatment yielded fewer annotated compounds than simple soaking (156 vs. 175 for CAVAF, 146 vs. 183 for CALF) (Figure 1). This reduction may reflect the degradation of certain low-abundance compounds that are unstable under the combined effects of ultrasonic cavitation and microwave heating [24]. At the same time, higher relative abundances of the principal flavonoid glycosides were observed under ultrasonic-microwave pretreatment. This pattern is typical of energy-assisted extraction. Ultrasound generates acoustic cavitation that disrupts cell walls and enhances mass transfer, while microwave provides rapid volumetric heating that accelerates the release of intracellular solutes [25]. 

*Citrus*-derived flavonoids, coumarins, and related polar metabolites have attracted considerable attention as natural antioxidants due to their capacity to scavenge free radicals and modulate redox homeostasis [1,26]. In the present study, the antioxidant activity of the hydrosol and still-bottom water differed markedly (Figure 6). The hydrosol displayed only modest radical scavenging activity and weak reducing power, which is consistent with their volatile chemical profile dominated by oxygenated monoterpenes (linalool, α-terpineol, and trans-geraniol) [13]. In contrast, the still-bottom water exhibited clear antioxidant activity in the DPPH, ABTS, and hydroxyl radical scavenging assays, with half-maximal inhibitory concentrations ranging from 3.338 to 4.873 mg/mL (DPPH), 2.384 to 2.683 mg/L (ABTS), and 0.1901 to 0.4853 mg/mL (hydroxyl radical). This radical scavenging capacity observed in the chemical assays is consistent with the high relative content of polar flavonoid glycosides retained in the residual aqueous fraction [27,28]. The persistently low reducing power of both fractions indicates that the compounds present are more effective as radical scavengers than as electron donors under the assay conditions employed. Previous work on phenolic extracts from *C. × aurantium* flowers has also reported strong scavenging of DPPH, ABTS, and hydroxyl radical [29]. Multiple studies on *Citrus* species have shown a positive association between total phenolic content and antioxidant capacity [30,31]. 

From a practical standpoint, the conventional discarding of still-bottom water after essential oil extraction represents a substantial loss of bioactive compounds. The present findings show that this residual fraction is chemically complex and possesses radical scavenging activity in chemical assays comparable to that of many conventional plant extracts. Recovery of the still-bottom water would therefore convert a current waste stream into a potential source of compounds with in vitro radical scavenging activity. At the same time, the results provide additional chemical compositional support for the food-medicine homology status of CAVAF and for the traditional practice of preparing aqueous infusions from the dried flower buds. It should be emphasized, however, that the data presented here are derived from a single commercial batch and analytical replicates only. Independent extraction batches and formal statistical evaluation will be required to confirm the observed trends. Moreover, in vitro chemical assays alone are quite limited; further biological and toxicological studies would be necessary before any practical application could be considered.

## 4. Materials and Methods

### 4.1. Plant Materials and Extraction Processes

The dried flower buds of *Citrus × aurantium* L. (voucher specimen number KUN1559515) and *Citrus × aurantium ‘Daidai’* (*Citrus aurantium* L. var. amara Engl., voucher specimen number IMC 0086979) used in this study were collected on 20 April 2022 from the sour-orange planting base of Sichuan Shugeng Agricultural Development Co., Ltd. (Guang’an, China) and belonged to the same commercial batch [13]. The materials were taxonomically authenticated by Professor Xianjian Zhou from the Sichuan Academy of Chinese Medicine Sciences. Both materials were ground to pass an 80-mesh sieve. For each variety, 15 g of powdered material was mixed with 210 mL of distilled water in a 500 mL round-bottom flask. Two pretreatment protocols were applied prior to hydrodistillation: (i) soaking-assisted extraction, in which the mixture was allowed to stand at room temperature for 1 h; and (ii) ultrasonic-microwave synergistic pretreatment, in which the flask was first treated in an ultrasonic cleaner (90 W, 7 min) and then irradiated in a microwave oven (280 W, 75 s). After the respective pretreatment, the mixture was subjected to conventional hydrodistillation using a standard steam distillation apparatus. The essential oil layer was discarded. The aqueous condensate was collected as the hydrosol in brown bottles, while the residual liquid remaining in the flask was vacuum-filtered to yield a clear filtrate designated as the still-bottom water. The hydrosol and still-bottom water were collected in glass bottles, stored at 4 °C in the dark, and analyzed within 1–2 days after extraction. Consequently, for each variety, four aqueous by-products were generated according to the combination of pretreatment method (soaking vs. ultrasonic-microwave) and fraction type (hydrosol vs. still-bottom water).

### 4.2. Analysis and Annotation of Chemical Components of Still-Bottom Water

HPLC-Q-TOF-HRMS analysis was performed by Ruisosi (Changzhou) Technology Co., Ltd. (Changzhou, China). Chromatographic separation was performed on a Waters BEH C18 column (1.7 μm, 2.1 × 50 mm). The mobile phase consisted of 0.1% formic acid in water (A) and 0.1% formic acid in acetonitrile (B). The column temperature was maintained at 40 °C and the flow rate was set at 0.4 mL/min. The gradient elution program was as follows: 0–2 min, 95% A and 5% B; 2–35 min, 2% A and 98% B; and 35–40 min, 95% A and 5% B.

Mass spectrometry was conducted on a quadrupole time-of-flight instrument equipped with an electrospray ionization source. In positive ion mode, the capillary voltage was 2 kV, the source temperature was 110 °C, the desolvation temperature was 400 °C, the nitrogen gas flow rate was 800 L/h, the mass range was 50–1200 *m*/*z*, and the collision energy was ramped from 20 to 40 V. In negative ion mode, the capillary voltage was adjusted to 1.5 kV, while all other parameters remained identical. 

Compound annotation was performed in Waters UNIFI using the 2023Z Traditional Chinese Medicine and Natural Products Database. Features were accepted when the mass error was within ±5.0 mDa. For each annotated peak, the observed retention time, molecular formula, adduct, observed *m*/*z* (precursor ion), mass error, and relative content (area normalization) are reported in Tables S1–S8. The reported assignments were regarded as putative annotations based on accurate-mass and database matching.

### 4.3. Evaluation of Antioxidant Activity

The antioxidant activities of the hydrosol and still-bottom water were evaluated via four complementary assays: DPPH radical scavenging, ABTS radical scavenging, hydroxyl radical scavenging, and reducing power determination. The still-bottom water was diluted to 10 mg/mL for the DPPH and ABTS radical scavenging assays, and to 1 mg/mL for the hydroxyl radical scavenging assay, while the hydrosol was tested undiluted in all cases. For the reducing power assay, the hydrosol was diluted to 50 mg/mL. Vitamin C was used as the positive control at 1 mg/L for the scavenging assays and at 5 mg/L for the reducing power assay. All measurements were performed in analytical triplicate.

The DPPH radical scavenging activity was assessed as previously reported [32] with minor modifications. A 0.1 mmol/L DPPH solution was freshly prepared in absolute ethanol and stored in the dark. Sample solution (100 µL) was mixed with DPPH solution (100 µL) (A_1_). To correct for sample background, sample solution (100 µL) was mixed with absolute ethanol (100 µL) (A_2_). The control consisted of absolute ethanol (100 µL) and DPPH solution (100 µL) (A_0_). After incubation at room temperature in the dark for 30 min, the absorbance was measured at 517 nm. The DPPH radical scavenging rate was calculated as

(1) $$DPPH\;scavenging\;rate\left(\%\right)=\frac{1-\left(A_{1}-A_{2}\right)}{A_{0}}\times 100,$$

where A_1_ is the absorbance of the sample group, A_2_ is the absorbance obtained when the DPPH solution in A_1_ is replaced by absolute ethanol, and A_0_ is the absorbance of the control.

The ABTS assay was performed following the procedure described in previous studies [11,33]. An ABTS stock solution was prepared by mixing equal volumes of 7.4 mmol/L ABTS solution and 2.6 mmol/L potassium persulfate and allowing the mixture to stand in the dark for 16 h. The stock was then diluted with absolute ethanol to an absorbance of 0.70 ± 0.02 at 734 nm. The control (Ac) contained absolute ethanol (200 µL) and ABTS working solution (800 µL), whereas the sample group (As) consisted of sample solution (200 µL) and ABTS working solution (800 µL). After incubation in the dark at room temperature for 30 min, the absorbance was recorded at 734 nm. The ABTS radical scavenging rate was calculated as

(2) $$ABTS\;scavenging\;rate\left(\%\right)=\frac{Ac-As}{Ac}\times 100,$$

where As and Ac represent the absorbance of the sample groups and the control, respectively.

The hydroxyl radical scavenging activity was evaluated using the Fenton reaction method [34]. Reagent solutions were 9 mmol/L FeSO_4_, 8.8 mmol/L H_2_O_2_, and 9 mmol/L salicylic acid (in absolute ethanol). The sample group (A_0_) contained sample solution (50 µL), H_2_O_2_ (50 µL), FeSO_4_ (50 µL), and salicylic acid (50 µL). The background group (A_1_) substituted distilled water for FeSO_4_, while the control (A_2_) substituted distilled water for the sample solution. After incubation in the dark at room temperature for 30 min, the absorbance was measured at 510 nm. The hydroxyl radical scavenging rate was calculated as

(3) $$Hydroxyl\;radical\;scavenging\;rate\left(\%\right)=\frac{1-\left(A_{0}-A_{1}\right)}{A_{2}}\times 100,$$

where A_0_, A_1_, and A_2_ represent the absorbance of the sample group, the sample background, and the control, respectively. Half-maximal inhibitory concentration was calculated using GraphPad Prism 9 by nonlinear regression (curve fit) with the log(inhibitor) versus normalized response-variable slope model.

The reducing power was determined based on a previous method [35] with slight adjustments. Sample solution (2 mL) was mixed with 0.2 mol/L phosphate buffer (pH 6.6, 2 mL) and 1% (*w*/*v*) potassium ferricyanide (2 mL). The mixture was incubated at 50 °C for 20 min, after which 10% (*w*/*v*) trichloroacetic acid (TCA) (2 mL) was added. After centrifugation at 10,000 rpm for 10 min, 2 mL of the supernatant was combined with 0.1% (*w*/*v*) FeCl_3_ (0.4 mL) and incubated at 50 °C for a further 10 min. Absorbance of the final test solution (As) was measured at 700 nm. The reducing power was expressed as the net absorbance:

(4) $$Reducing\;power\left(Abs\right)=As-Ac,$$

where As is the absorbance of the sample and Ac is the absorbance of a blank prepared by substituting distilled water for the sample.

### 4.4. Statistical Analysis

Screening and annotation of chemical constituents, as well as data processing, were performed using the Waters UNIFI database and Microsoft Excel 2016 software. Venn diagrams were generated with the online tool available at https://bioinfogp.cnb.csic.es/tools/venny/index.html (accessed on 3 January 2024). Hierarchical cluster plots were prepared with standard bioinformatics tools. Histograms and principal component analysis were carried out with Origin 2018. 

The HPLC-Q-TOF-HRMS analysis used for compound annotation was performed only once for each of the four still-bottom water samples. For the antioxidant activity assays, three independent extractions were carried out for each condition, and the measurements were performed on the resulting extracts. Error bars in the corresponding figures represent the standard deviation of these three measurements. The statistically significant difference was assessed using Duncan’s multiple range test within a one-way ANOVA framework in SPSS Statistics 25.

## 5. Conclusions

Still-bottom water recovered after hydrodistillation of CAVAF and CALF is rich in flavonoid glycosides and displays appreciable radical scavenging activity in the chemical assays employed. In the present single-batch comparison, botanical variety and the pretreatment applied before distillation appeared to influence both the metabolite profile and the measured antioxidant capacity in a consistent manner. These findings provide chemical compositional support for the food-medicine homology status of CAVAF and indicate that the residual aqueous fraction, currently discarded, constitutes a recoverable source of compounds with in vitro radical scavenging capacity.

## Figures and Tables

**Table 1:** Distribution of common compound types identified in still-bottom water obtained under different pretreatments and from different varieties.

Compound Type	Number of Compounds
Flavonoids	42
Coumarins	10
Phenylpropanoids	8
Organic acids	8
Alkaloids	7
Anthraquinones	7
Terpenoids	7
Others	7
Limonoids	5
Phenols	3
Glycosides	2
Amino acids	2
Aldehydes	1
Alcohols	1
Caffeoyl derivatives	1
Bufogenins	1
Steroids	1
Phthalates	1
Cerebrosides	1
Xanthones	1
Quassinoids	1
Phenylethanoid glycosides	1
Total	118

**Table 2:** Distribution of unique compound types identified in still-bottom water obtained under different pretreatments and from different varieties.

Compound Type	Soaking CAVAF	Ultrasonic-Microwave CAVAF	Soaking CALF	Ultrasonic-Microwave CALF
Flavonoids	–	4	3	2
Terpenoids	1	–	2	1
Alcohols	1	–	–	–
2-(2-Phenylethyl)chromones	1	–	–	1
Phenylpropanoids	–	1	2	–
Phenanthrenes	–	1	2	–
Amino acids	–	–	1	–
Anthraquinones	–	–	1	–
Aldehydes	–	–	1	–
Bufadienolides	–	–	1	–
Organic acids	–	–	1	–
Alkaloids	–	–	–	1
Coumarins	–	–	–	1
Triglycerides	–	–	–	1
Others	2	–	1	1
Total	5	6	15	8

## Data Availability

The original contributions presented in this study are included in the article/Supplementary Materials. Further inquiries can be directed to the corresponding authors.

## Supplementary Materials

The following supporting information can be downloaded at https://www.mdpi.com/article/10.3390/plants15172583/s1, Figure S1: Total ion chromatograms (TIC) of still-bottom water from CAVAF under different pretreatments and ionization modes. (a) Soaking, positive ion mode. (b) Soaking, negative ion mode. (c) Ultrasonic–microwave, positive ion mode. (d) Ultrasonic–microwave, negative ion mode; Figure S2: Total ion chromatograms (TIC) of still-bottom water from CALF under different pretreatments and ionization modes. (a) Soaking, positive ion mode. (b) Soaking, negative ion mode. (c) Ultrasonic–microwave, positive ion mode. (d) Ultrasonic–microwave, negative ion mode; Table S1: Analysis of the chemical components of the soaking‑derived still‑bottom water of CAVAF (positive ion mode); Table S2: Analysis of the chemical components of the soaking‑derived still‑bottom water of CAVAF (negative ion mode); Table S3: Analysis of the chemical components of the ultrasonic-microwave synergistic-assisted still-bottom water of CAVAF (positive ion mode); Table S4: Analysis of the chemical components of the ultrasonic-microwave synergistic-assisted still-bottom water of CAVAF (negative ion mode); Table S5: Analysis of the chemical components of the soaking-derived still-bottom water of CALF (positive ion mode); Table S6: Analysis of the chemical components of the soaking-derived still-bottom water of CALF (negative ion mode); Table S7: Analysis of the chemical components of the ultrasonic-microwave synergistic-assisted still-bottom water of CALF (positive ion mode); Table S8: Analysis of the chemical components of the ultrasonic-microwave synergistic-assisted still-bottom water of CALF (negative ion mode); Table S9: Analysis of antioxidant activities of hydrosol and still-bottom water derived from CAVAF and CALF.

## Abbreviations

The following abbreviations are used in this manuscript:

CAVA	*Citrus aurantium* L. var. amara Engl.
CAL	*Citrus* × *aurantium* L.
CAVAF	the dried flower buds of *Citrus aurantium* L. var. amara Engl.
CALF	the dried flower buds of *Citrus* × *aurantium* L.
HPLC-Q-TOF-HRMS	high-performance liquid chromatography-quadrupole-time of flight high-resolution mass spectrometry
DPPH	1,1-diphenyl-2-picrylhydrazyl radical
ABTS	2,2′-Azino-bis(3-ethylbenzothiazoline-6-sulfonic acid) diammonium salt
PCA	principal component analysis
TIC	total ion current
