# Supplementary material for: Impact of Two Pretreatment Methods on the Chemical Profile of Still-Bottom Water and the Antioxidant Activities of Hydrosol and Still-Bottom Water from Citrus × aurantium ‘Daidai’ and Citrus × aurantium L. Dried Flower Buds
Source: Plants (Basel). 2026 Aug 25;15(17):2583. doi: 10.3390/plants15172583 (PMC13567354; doi:10.3390/plants15172583)
Supplement: Supplementary file 1 [file plants-15-02583-s001.zip › plants-4509198-Supplementary figures.pdf]

Supplementary figures

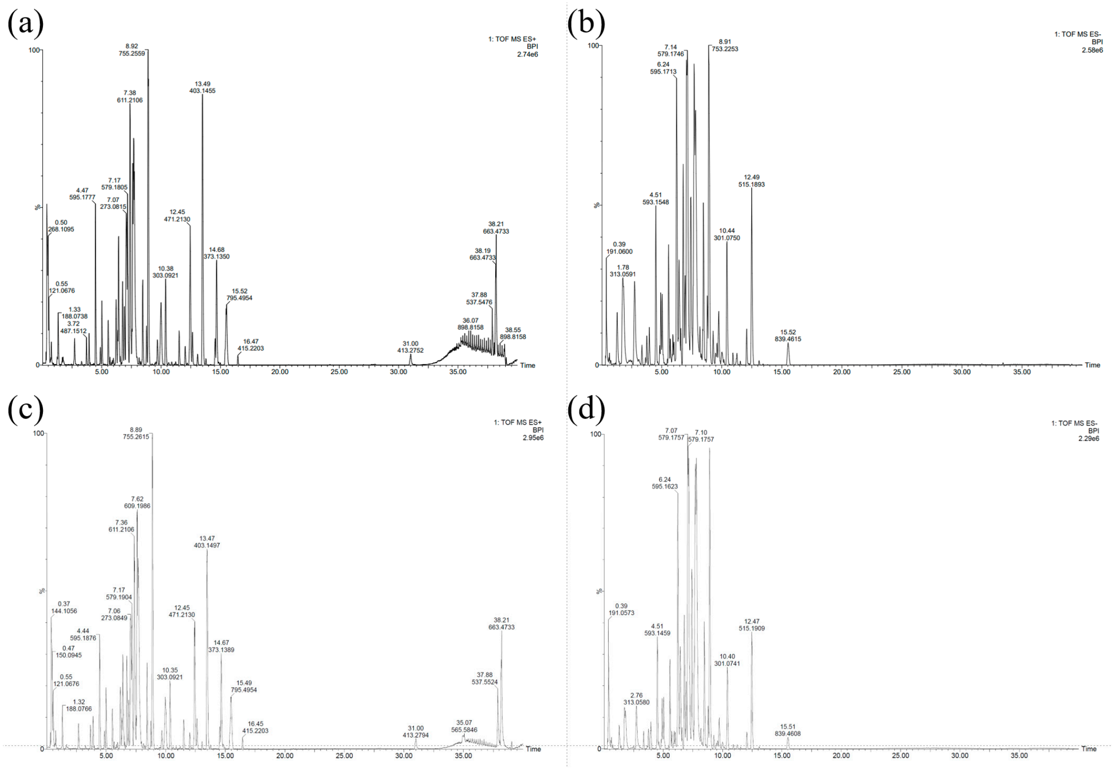

Supplementary Figure S1 Total ion chromatograms (TIC) of still-bottom water from CAVAF under different pretreatments and ionization modes. (a) Soaking, positive ion mode. **(b)** Soaking, negative ion mode. **(c)** Ultrasonic–microwave, positive ion mode. **(d)** Ultrasonic–microwave, negative ion mode.

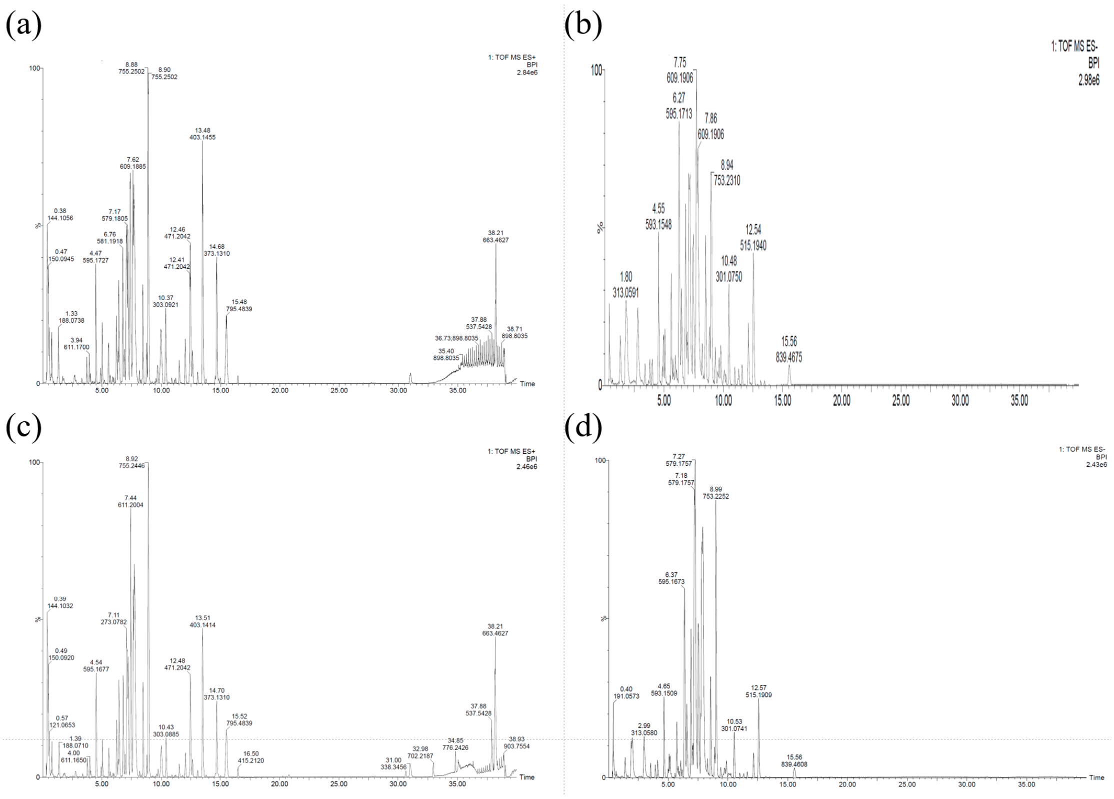

Supplementary Figure S2 Total ion chromatograms (TIC) of still-bottom water from CALF under different pretreatments and ionization modes. (a) Soaking, positive ion mode. **(b)** Soaking, negative ion mode. **(c)** Ultrasonic–microwave, positive ion mode. **(d)** Ultrasonic–microwave, negative ion mode.
